# Predicting the occurrence of surgical site infections using text mining and machine learning

**DOI:** 10.1371/journal.pone.0226272

**Published:** 2019-12-13

**Authors:** Daniel A. da Silva, Carla S. ten Caten, Rodrigo P. dos Santos, Flavio S. Fogliatto, Juliana Hsuan

**Affiliations:** 1 Industrial Engineering Department, Universidade Federal do Rio Grande do Sul, Porto Alegre, Brazil; 2 Hospital de Clinicas de Porto Alegre, Porto Alegre, Brazil; 3 Copenhagen Business School, Copenhagen, Denmark; University of Pennsylvania, UNITED STATES

## Abstract

In this study we propose the use of text mining and machine learning methods to predict and detect Surgical Site Infections (SSIs) using textual descriptions of surgeries and post-operative patients’ records, mined from the database of a high complexity University hospital. SSIs are among the most common adverse events experienced by hospitalized patients; preventing such events is fundamental to ensure patients’ safety. Knowledge on SSI occurrence rates may also be useful in preventing future episodes. We analyzed 15,479 surgery descriptions and post-operative records testing different preprocessing strategies and the following machine learning algorithms: Linear SVC, Logistic Regression, Multinomial Naive Bayes, Nearest Centroid, Random Forest, Stochastic Gradient Descent, and Support Vector Classification (SVC). For prediction purposes, the best result was obtained using the Stochastic Gradient Descent method (79.7% ROC-AUC); for detection, Logistic Regression yielded the best performance (80.6% ROC-AUC).

## 1. Introduction

Surgical Site Infections (SSIs) are one of the predominant types of infection in Brazilian hospitals [1]. About one in thirty "clean" surgeries will suffer from complications due to SSIs. The rate is significantly higher if we consider "dirty" (i.e. contaminated), emergency, and prolonged surgeries, or procedures performed on patients with clinical comorbidities [2]. SSIs are also among the most frequent Adverse Events (AEs) reported on hospitalized patients, causing a substantial increase in mortality, re-hospitalization rates, and care costs [2,3].

Traditional methods for the prevention and detection of infections typically use resources (mostly human) in an intensive and time-consuming way. Computerized techniques, mainly based on Artificial Intelligence, may provide expedite and cost-efficient alternatives to the analysis of infections [4–6]. For that, it is necessary to verify the applicability of those techniques in the detection of AEs and control of hospital infections, particularly in large scale, data-rich environments such as the Brazilian healthcare system [7].

Health surveillance has been described as an essential part of infection prevention and control programs due to its ability to promote a decrease in infection rates [8–10]. In healthcare institutions, patient information is stored mainly in the form of narrative texts and clinical reports [11]. Passive search for infections is usually carried out analyzing spontaneous reports made by health professionals (i.e. healthcare providers report clinical signs and a possible infection diagnosis in the patient’s medical record, but not necessarily make the statement of a hospital infection to the surveillance entities). However, most mild and self-limiting infections are likely to remain unreported. On the other hand, surveillance and the active search for infections usually do not provide real-time information, since data collection, analysis and feedback traditionally rely on time and resource consuming methods [12]. Data Mining (DM) and Machine Learning (ML) techniques provide an alternative for that.

The use of DM to support health surveillance has been reported in the literature [13–15]. When applied to unstructured textual data, DM is referred to as Text Mining (TM); DM and TM share the same process and goal of identifying non-trivial patterns in data that are both meaningful and useful to users [16]. Both groups of techniques often use ML algorithms [17,18], enabling the prediction and classification of new records based on knowledge gathered from existing records.

There is some evidence in the literature reporting the successful use of TM and ML methods in the analysis of events that cause harm to patients; see [4–6]. Machine learning has been shown to be an effective tool for predicting infections [19,20]. In the same way, significant advances were also reported on the subject of adverse events’ extraction and detection using free text to improve patients’ diagnosis [21,22]. However, there is a gap in the literature regarding the joint application of TM and ML to predict SSI mining textual records of surgical descriptions, which we aim to bridge with our study.

## 2. Materials and methods

TM methods were used to process surgeries’ and post-operative patients’ records of a Brazilian hospital aiming to set the best practices for predicting and detecting SSIs using ML algorithms. An optimization of hyperparameters has also been performed for each algorithm. We analyzed a dataset comprised of textual descriptions of surgeries and post-operatives patients’ records up to 30 days after the procedure.

The dataset was obtained from Hospital de Clínicas de Porto Alegre (HCPA), an 842-bed, tertiary care teaching hospital located in the city of Porto Alegre, Brazil. The hospital is deemed best in the country in its category, providing average and high complexity care through the Brazilian Unified Health System (SUS). There are 14 surgical specialties considered in the analysis.

The 30-day observation period established in this study to monitor the occurrence of post-surgical infections is grounded on empirical evidence as shown in Table 1, which was obtained mining a database of surgeries performed by each specialty in the past 5 years.

**Table 1:** List of surgical specialties and associated post-operative length-of-stay.

*Specialty*, *i*	*n*_*i*_	*t*_*i*_
1. Pediatrics	2,963	58.5
2. Colorectal	2,516	32.8
3. Neurosurgery	2,392	27.1
4. Digestive System	9,930	26.6
5. Urology	11,136	22.0
6. Vascular	3,435	21.9
7. Plastic	1,663	21.8
8. Thoracic	3,213	20.6
9. General	9,294	15.7
10. Orthopedics and Traumatology	6,468	13.0
11. Gynecology and Obstetrics	5,398	8.8
12. Otorhino	5,652	8.5
13. Oral and Maxillofacial	278	7.5
14. Mastology	2,034	3.0

In Table 1, *n*_*i*_ denotes the number of records found in the 5-year period, and *t*_*i*_ denotes the average number of postoperative days of hospital stay demanded by specialty *i*. Statistic *t*_*i*_ is a weighted average, which considered the average number of postoperative hours of hospital stay demanded by each type of procedure within specialty *i* and their frequency of occurrence in the database. Most specialties require post-operative stays shorter than 30 days (the overall mean is 20.5 days), justifying the observation period established here.

**Fig 1:**
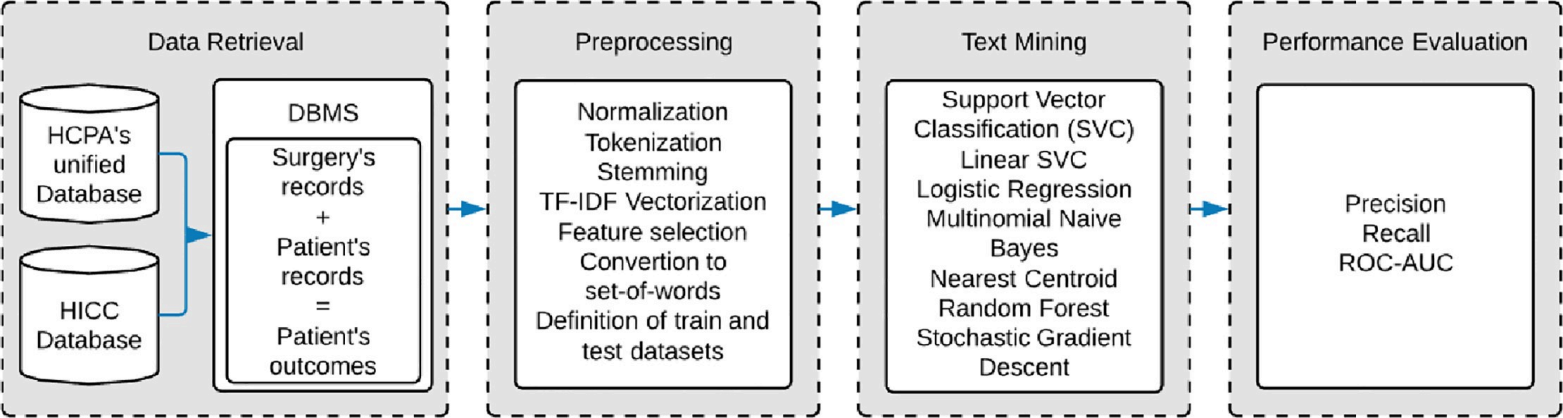
Overview of the proposed method.

Data were used to train and compare classification algorithms and text preprocessing techniques. The study was conducted in four stages (Fig 1), which were adapted from [23].

Data were managed using PostgreSQL 9.6 [24]. Text preprocessing was carried out in Python 3.5 [25], which was also used to run TM and ML methods, and evaluate their performance. Python provides libraries to support processing of records, information retrieval, application and validation of methods; we used the NLTK [26] and scikit-learn [27] libraries.

In the *data retrieval* stage, we retrieved textual information on surgeries and patients’ post-operative records from HCPA’s unified database, which was then combined with inputs from the hospital’s Internal Committee for Infection Control (ICIC). The committee retrospectively reviews records identifying the ones that led to infections, following an active search strategy. Records reviewed are manually selected among those more likely to display a patient infection outcome; i.e. patients to whom antibiotics were prescribed, cases in which positive wound cultures are reported or those associated to patients displaying signs and symptoms of fever, hyperemia or presence of secretion in their evolutions, older or obese patients, and those carrying chronic diseases such as diabetes. The committee reviewed the selected post-operative records and reached a conclusion, assigning one of two possible outcomes (patient infected or not infected).

There are two parts to the text records used here: (*i*) a technical description of the surgical procedure, and (*ii*) follow-ups on the evolution of patients during hospitalization and consultations, up to 30 days after surgery. Records analyzed were written in Brazilian Portuguese. The occurrence of infections is reported in both parts by those providing care to patients, leading to a highly unbalanced dataset: 1.2% of the records report infections in part (*i*), while 1.6% report infections in part (*ii*). Records reporting infections on the 31^st^ day after surgery or later were disregarded, as well as those of patients who had more than one surgery performed on the same day with different infection outcomes.

The response variable used in the text mining step to obtain predictions of outcomes was the “gold standard” established by the ICIC, and not the caretakers’ reports. Thus, we may have situations in which the surgery was reported infected by caretakers, but no infection was reported by them during the post-operative period, although the case was considered infected since that was the conclusion issued by the ICIC. Note that the ICIC issued one conclusion per case; thus, whenever the ICIC concluded that a case was infected, the conclusion was valid for both surgery and post-operative descriptions.

The second stage is the *Text Mining*. There are six steps in this stage: normalization, tokenization and stemming, vectorization, feature selection, conversion to set-of-words, and definition of the train and test sets. Textual description of cases was obtained in the previous stage in *comma-separated values* (.csv) format and inserted into the PostgreSQL database. The dataset was structured with three fields of information: (*i*) the outcome of a binary variable representing the final status of the patient (1 = infected or 0 = not infected) obtained from the ICIC; (*ii*) free-form text entered directly by healthcare providers describing the surgery; and (*iii*) free-form text describing the post-operative record, also entered by caretakers. Data from the second and third fields were treated in the pre-processing module. Numerical entries were excluded from fields (*ii*) and (*iii*).

In the normalization step, stop words and punctuation were removed and the text was rewritten with no capital letters. In the tokenization step, continuous text was reduced to tokens, which are linguistic units such as words and sentences [28]. Morphological normalization was also carried out with words reduced to root form such that gender and grade information was excluded. After this step, each record was comprised of a set of tokens, delimited by blank spaces. Once tokens are identified, and prior to the feature selection step, features must be defined. In general, an *n-*gram is a sequence of *n* tokens [29]. In this work, we used unigrams, bigrams, and trigrams as features.

In the feature selection step, features were ranked according to two indices. The first is based on the *χ*^2^ test, commonly used to verify the independence between a pair of events; in the context of feature selection, we test the occurrence of features in classes and their dependence using Eq (1).

$$\chi^{2}(d,t,c)=\sum_{e_{t}\in \{0,1\}}\sum_{e_{c}\in \{0,1\}}\frac{{\left(N_{e_{t}e_{c}}-E_{e_{t},e_{c}}\right)}^{2}}{E_{e_{t},e_{c}}}$$ (1)

where *N* and *E* are the observed and expected occurrence frequencies in document *d*, *e*_*t*_ is a binary variable indicating if feature *t* occurs in *d*, and *e*_*c*_ is a binary variable indicating if *d* is in class *c*. For the independence hypothesis to hold, $N_{e_{t}e_{c}}$ and $E_{e_{t},e_{c}}$ should converge to 0.5; when that is not the case and *χ*^2^ values are large, feature *t* should be selected [30].

The second feature selection index is based on F-value, which is calculated as follows [31]:

$$F\;value(t)=\frac{{\left(X_{t}^{inf}-{X´}_{t}\right)}^{2}+{\left(X_{t}^{n\_inf}-X_{t}\right)}^{2}}{\frac{1}{n_{inf}-1}\sum_{k=1}^{n_{inf}}{\left(X_{k,t}^{inf}-X_{t}^{inf}\right)}^{2}+\frac{1}{n_{n\_inf}-1}\sum_{k=1}^{n_{n\_inf}}{\left(X_{k,t}^{n\_inf}-X_{t}^{n\_inf}\right)}^{2}}$$ (2)

where *X*_*t*_, $X_{t}^{inf}$ and $X_{t}^{n\_inf}$ is the average of the *t*-th feature in the complete, infected, and non-infected datasets, respectively; $X_{k,t}^{inf}$ is the *t*-th feature of the *k-*th infected instance, and $X_{k,t}^{n\_inf}$ is the *t*-th feature of *k-*th non-infected instance; *n*_*inf*_ is the number of infected instances, and *n*_*n*_*inf*_ is the number of non-infected instances. Eq (2) gives a measure of discrimination between the two sets (infected and non-infected); whenever the F-value of a feature is greater than a threshold value the feature is inserted into the selected feature space; otherwise, it is removed from that space. High F-values indicate discriminating features [31].

Features retained were those above a given percentile of largest values; percentile and index chosen varied according to the Text Mining method tested. Indices were chosen based on their good performance in previous studies [5,23], and adequacy to the case under analysis.

In the conversion to set-of-words step, the occurrence of features (selected in the previous step) in records is evaluated regarding a given indicator, and results are organized in a matrix. Text records are listed in matrix rows and selected features in matrix columns. Two indicators were tested here: term frequency (TF), and term frequency-inverse document frequency (TF-IDF). TF gives the frequency in which a feature appears in a record. TF-IDF reflects the importance of a feature in a record from a collection of records, increasing proportionally to the feature’s frequency in a record, but being compensated by the feature’s frequency in the collection of records, as given next [32]:

$$TFIDF(t,d,D)=\frac{f(t,d)}{max\{f(t,d):t\in d\}}\times \log\frac{\left|D\right|}{\left|\left\{d\in D:t\in d\right\}\right|}$$ (3)

where *t* denotes the feature, *d* denotes the record, *D* is the total number of records considered, and *f*(*t*,*d*) is the number of occurrences of feature *t* in record *d*.

*TFIDF* values for each matrix term were normalized using the unitary Euclidian norm [32], as follows:

$$v_{norm}=\frac{v}{{||v||}_{p}}=\frac{v}{\sqrt{v_{1}^{p}+v_{2}^{p}+\cdots +v_{n}^{p}}}$$ (4)

where **v**_*norm*_ is the normalized vector, **v** is the vector to be normalized and ||**v**||^*p*^ is the norm used to promote normalization, such that *p* = 1 for $l_{1}norm$ and *p* =2 for $l_{2}norm$ [33]. Large values of *TFIDF* are obtained whenever a term displays high frequency in a document and low frequency in the complete set of documents.

The last step of the *pre-processing* stage is the definition of training and testing sets, in which the set-of-words is divided to allow *k*-fold cross-validation. We divided the dataset into *k* mutually exclusive subsets of equal size, and used one subset for testing and *k*−1 subsets for parameter estimation. The process was carried out *k* = 10 times alternating the test subset, and performance statistics were calculated from the results [34].

In the *Machine Learning* stage, we tested different supervised classification algorithms. In supervised learning the outcome of each analyzed record in known beforehand. Records are in the format (**x**,*y*), where **x** is the vector of features defined in the preprocessing stage and *y* is a binary class identifier, with outcomes 0 for not infected (or clean), and 1 for infected.

We tested the performance of ML algorithms used in similar studies; they are: Support Vector Machines (SVM) [13,14,35–45], Logistic Regression [37,42], Naive Bayes [35,38,43,44,46], Boosted Trees [38], Random Forest [38], and Nearest Neighbors [38]. The dataset was split into training and testing sets. Due to the highly unbalanced dataset, we used a stratified (*k*−1) cross-validation strategy, preserving the percentage of samples in each class in each fold, with *k* set to 10 [47]; that means the learning process is executed 10 times in different training sets, and the average of 10 scores is used to obtain an overall accuracy estimate. The goal was to improve the algorithms’ performance on the classification of records resulting in infection. Records were randomly picked in the training set such that each class is represented in the same proportions observed in the complete sample of records (~98.6% clean and ~1.4% infected outcomes).

Each ML algorithm has parameters that are not directly learned by the classifier; e.g., *C*, kernel, and *γ* in SVM [48]. Parameters’ definition was performed through a random search in a grid of parameters [49]. The search had the objective of finding the best combination of parameters to maximize the Area Under the Curve (AUC) of the Receiver Operating Characteristic (ROC) [50]. The grid search was also used to select the percentile and metric (*χ*^2^ and *F*-score) for attribute selection, and norm for TF-IDF (*l*1 or *l*2).

The final stage in our proposed method is *performance evaluation*. For that, we analyzed precision, sensitivity, ROC-AUC, and confusion matrices. These indicators are suitable for classification problems with unbalanced datasets, being used several similar studies reported in the literature [13,14,43–46,51,35–42].

## 3. Results

The original database was comprised of 27,648 surgical descriptions and 15,714 post-operative records (the use of the dataset was approved by HCPA’s Ethics Committee under project number CAAE 33705014.8.0000.5327). HCPA’s Ethics Committee is coordinated by Drs. Temis Maria Felix and Marcia Mocellin Raymundo; the complete list of Committee members is available at https://www.hcpa.edu.br/downloads/pesquisa/ato_n_188-2019.pdf. After excluding empty records and those that did not fit the criteria of the study, the number of records was reduced to 15,479 surgical descriptions and 12,637 post-operative records, with 98.6% of the records negative and 1.4% positive on average, according to Table 2. Table 3 provides a descriptive view of the final dataset (datasets and codes used in this analysis are given in S1 Supplement). Some remarks are noteworthy. Records in the database cover an 8-month period starting in 12/2015. During that period: (*i*) 27,648 surgical descriptions were made; of those, the ICIC audited a sample and detected 247 infections; and (*ii*) 15,714 post-operative descriptions were made; of those, the ICIC audited a sample and detected 233 infections. We excluded records of patients who had more than one surgery in the same day and only one of them was infected, since there is a single post-operative record in such situation.

**Table 2:** SSI database analyzed in this study.

	Prediction			Detection		
*Description*	Infected surgeries	Clean surgeries	Total	Infected surgeries	Clean surgeries	Total
Initial sample	247	27,401	27,648	233	15,481	15,714
Empty records	-29	-12,103	-12,132	-2	-3,037	-3,039
Records of patients that had more than one surgery, one of which was reported clean	-4	-7	-11	-3	-9	-12
Infections reported more than 30 days after surgery	-26	0	-26	-26	0	-26
Records used in the study (final sample)	188 (1.21%)	15,291 (98.79%)	15,479 (100%)	202 (1.6%)	12,435 (98.4%)	12,637 (100%)

**Table 3:** Descriptive view of the dataset.

Characteristic	Value
Number of patients	12,483
Mean (and SD) of patients’ age	48.31 (22.03)
Average number of surgeries per patient	1.24
Female patients	7,107
Mean (and SD) of female patients’ age	47.13 (20.44)
Male patients	5,376
Mean (and SD) of male patients’ age	49.88 (23.87)
Number of surgical procedures	18,062
Elective procedures	13,027
Urgent procedures	3,239
Emergency procedures	1,796
Average size (and SD) of surgical team	5.84 (2.81)

In results to follow, text mining classifiers were used in the two parts of the dataset (surgical descriptions and post-operative descriptions) separately. We refer to results in the first part (surgical descriptions) as *prediction*, and to results in the second part (post-operative descriptions) as *detection*. In both cases, TM pre-processing and ML algorithms were used to classify cases as clean or infected.

**Fig 2:**
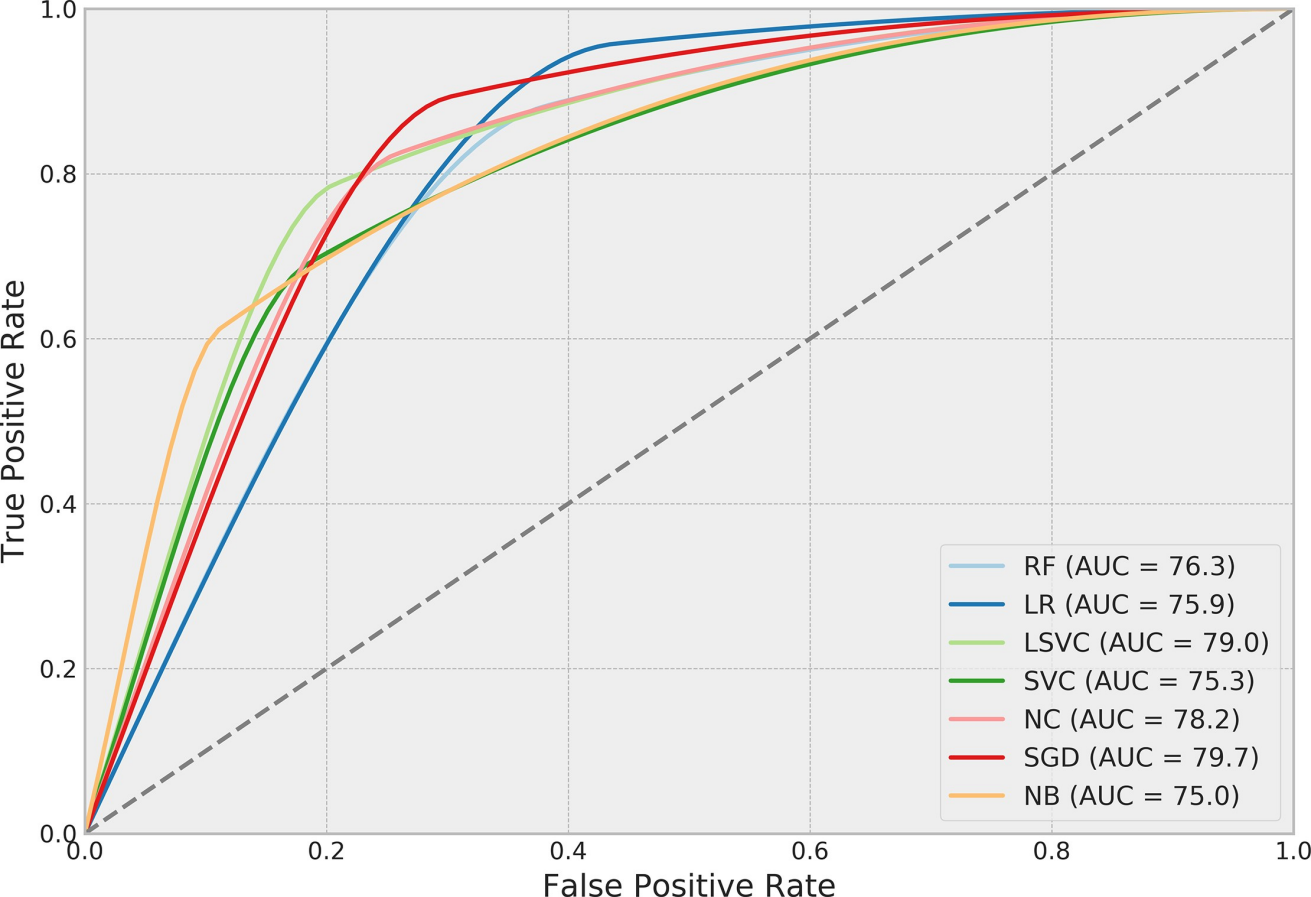
ROC-AUC performance of algorithms in predicting SSIs.

**Fig 3:**
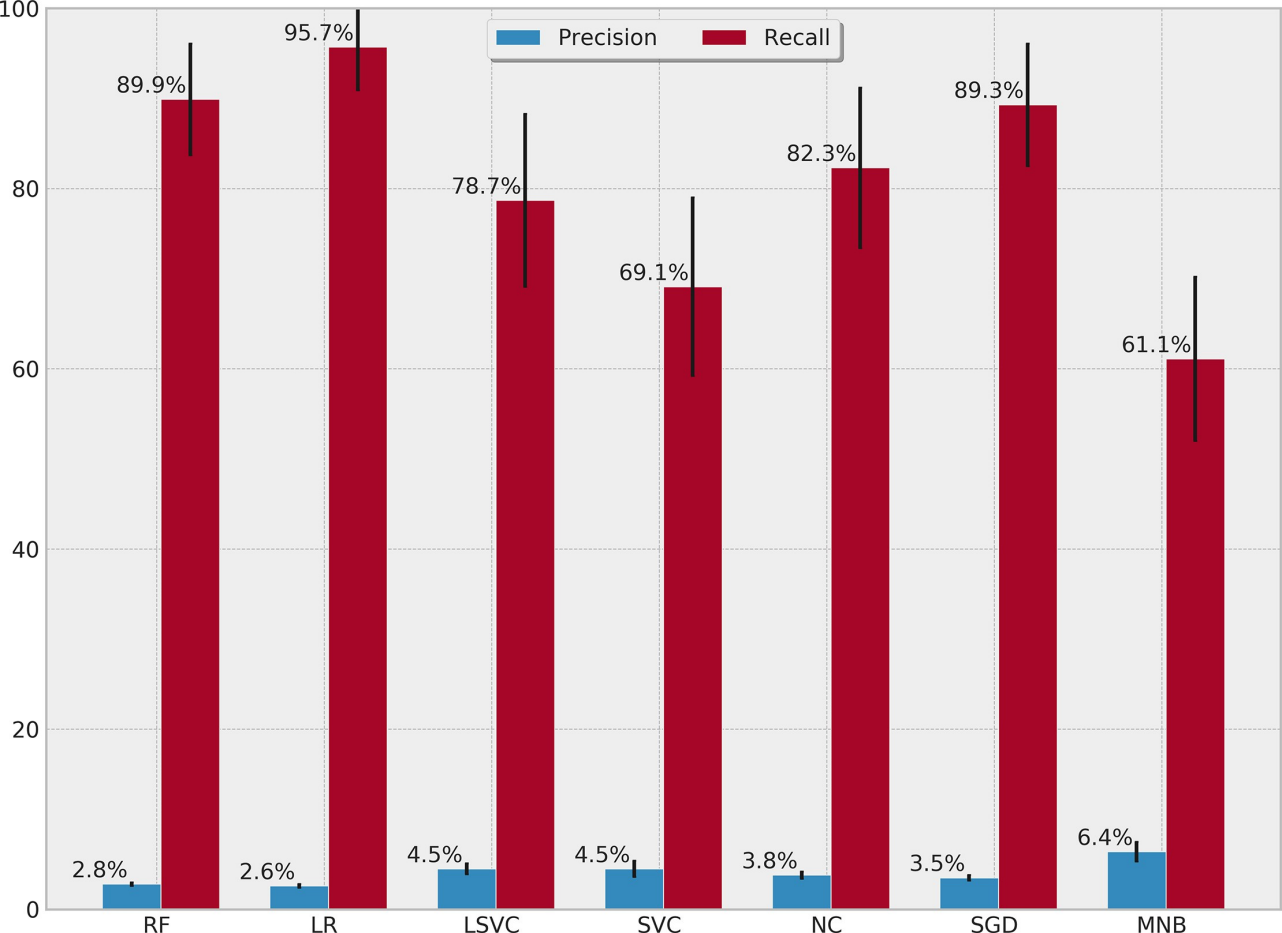
Precision-recall percentages and boxplots for surgical descriptions.

**Fig 4:**
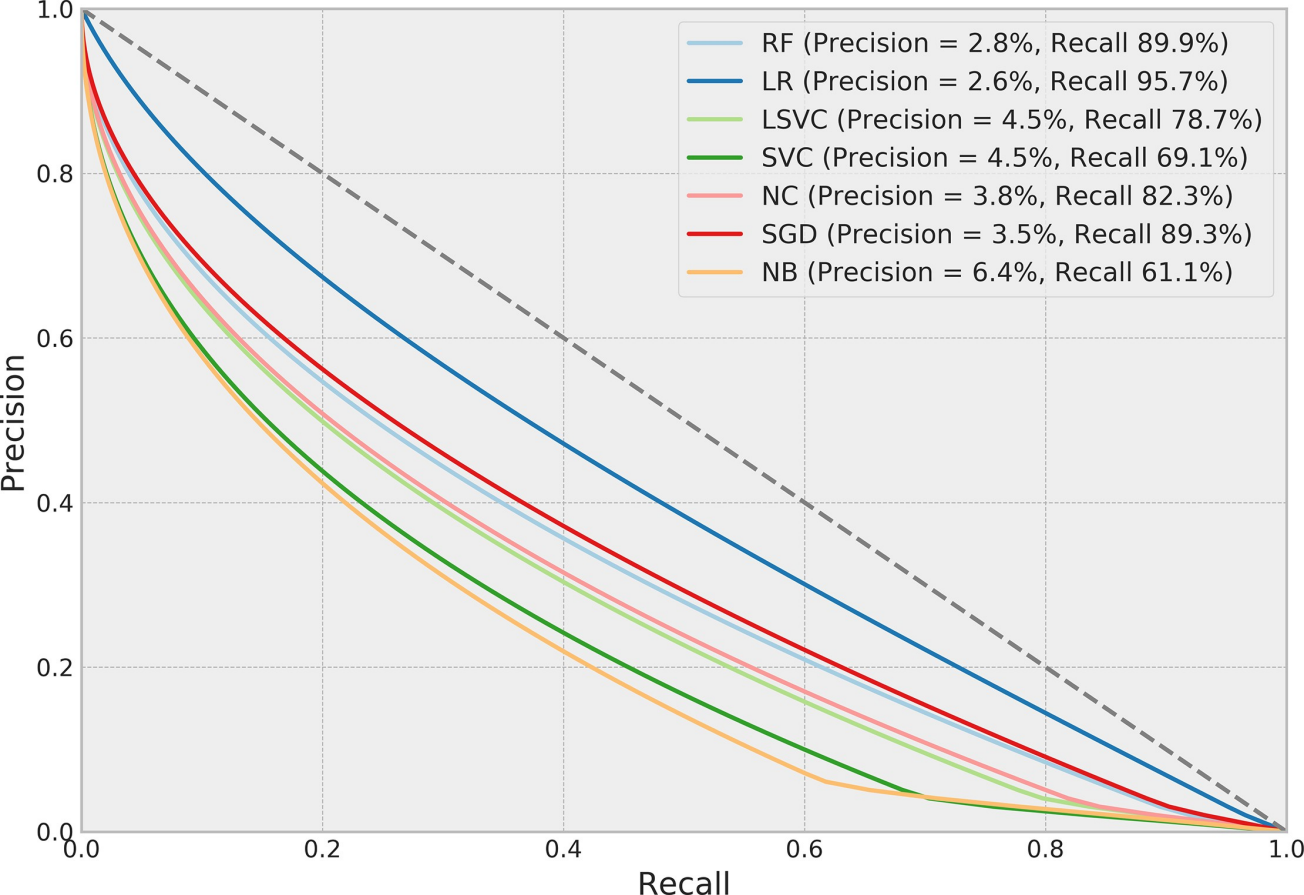
Precision-recall curves of methods tested for predicting SSIs.

Table 4 presents the performance of each ML algorithm in predicting infections and the respective TM settings to achieve the results. ROCs for the prediction algorithms are shown in Fig 2, Precision-Recall boxplots in Fig 3 and Precision-Recall curves for all tested methods in Fig 4. The best performance considering the relationship between true positives and false positives represented by the area under the ROC curve was obtained by the Stochastic Gradient Descent (SGD) method. The best result was achieved using 75% of the terms selected by the *χ*^2^ test in a set of features without normalization, assigning a weight of 0.01 to the negative (clean) class. Pre-processing strategies for each method were determined from a grid of parameter options through random search. Chosen Feature Selection method, Percentile, Transformation, Normalization, and Class_Weight options were those yielding the best ROC-AUC.

**Table 4:** Algorithms’ performance in predicting SSI.

						ROC-AUC	
Method	T	P	FS	N	CW	Mean	SD
Random Forest (RF)	TF-IDF	85%	F	*l*1	1/0.01	76.3%	3.3%
Logistic Regression (LR)	TF-IDF	55%	F	*l*1	1/0.005	75.9%	2.5%
Linear SVC (LSVC)	TF	85%	F	*l*1	1/0.005	79.0%	4.7%
SVC	TF	10%	F	*l*2	1/0.001	75.3%	4.6%
Nearest Centroid (NC)	TF	20%	*χ*^2^	*l*1	-	78.2%	4.3%
SGD	TF	75%	*χ*^2^	-	1/0.01	79.7%	3.3%
M-Naive Bayes (MNB)	TF	40%	*χ*^2^	-	20/80	75.0%	4.6%

T: Transformation; P: Percentile; FS: Feature Selection; N: Normalization; CW: Class_Weight / Prior Probability; SD: Standard Deviation

SGD classification with the pre-processing settings displayed in Table 4 reached a mean of 79.7% ROC-AUC (SD = 3.3%), mean sensitivity of 89.3% (SD = 6.8%) for positive classes, and 65% (SD = 1.4%) for negative classes. Considering the unbalance between classes, mean precision values obtained were 3.5% (SD = 0.3%) for the positive class and 99.8% (SD = 0.1%) for the negative class.

**Fig 5:**
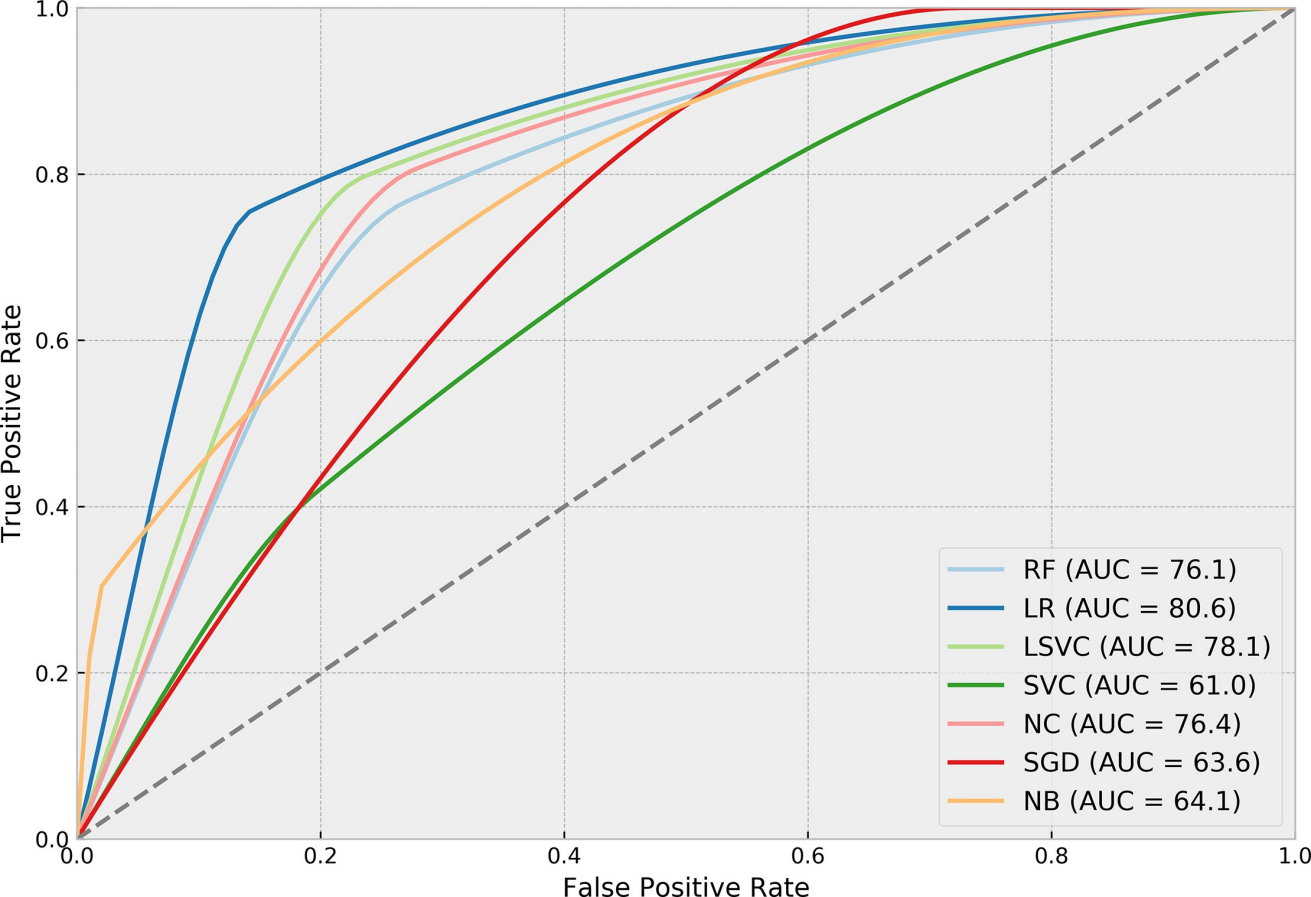
ROC-AUC performance of algorithms in detecting SSIs.

**Fig 6:**
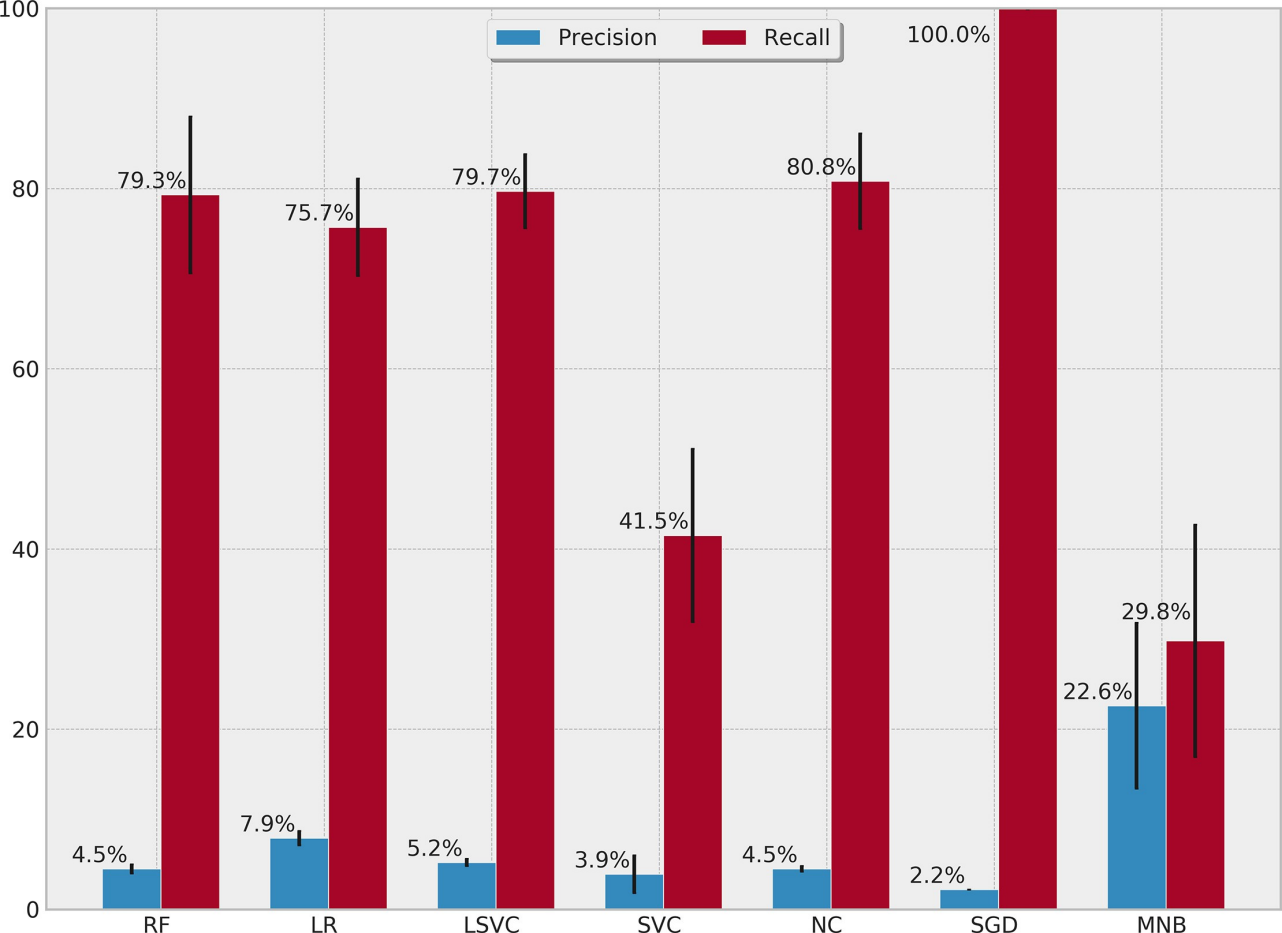
Precision-recall percentages and boxplots for post-operative notes.

**Fig 7:**
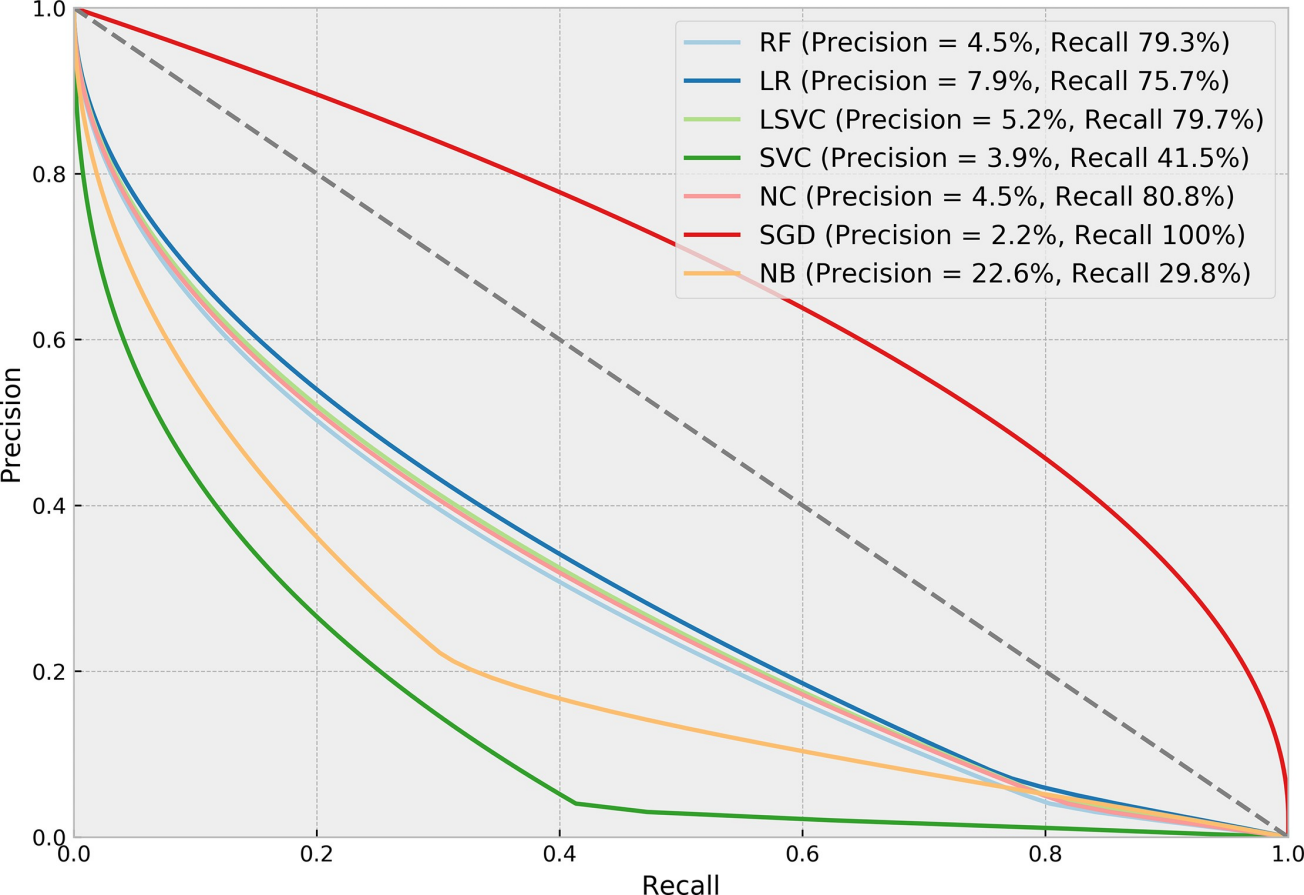
Precision-recall curves of methods tested for detecting SSIs.

Table 5 presents the performance of each ML algorithm in detecting infections and the corresponding TM settings. ROCs for the detection algorithms are shown in Fig 5, Precision-Recall boxplots in Fig 6 and Precision-Recall curves for all tested methods in Fig 7. Logistic regression was the method yielding the best results considering the relationship between true positives and false positives. This result was achieved using 40% of the terms selected from the *χ*^2^ test, in a set of TF-IDF terms normalized using the norm *l*1, with a weight of 0.01 for the negative class. The method yielded an ROC-AUC of 80.60% (SD = 2.4%), mean sensitivity of 75.7% (SD = 5.4%) for positive classes, and 85.5% (SD = 1.5%) for negative classes. Mean precision values were 7.9% (SD = 0.8%) for positive class, and 99.5% (SD = 0.1%) for negative class.

**Table 5:** Algorithms’ performance in detecting SSI.

						ROC-AUC	
Method	T	P	FS	N	CW	Mean	SD
Random Forest (RF)	TF	20%	*χ*^2^	*l*2	1/0.01	76.1%	3.4%
Logistic Regression (LR)	TF-IDF	40%	*χ*^2^	*l*1	1/0.01	80.6%	2.4%
Linear SVC (LSVC)	TF-IDF	45%	*χ*^2^	*l*1	1/0.01	78.1%	2.5%
SVC	TF	25%	F	*l*2	1/0.1	61.0%	6.3%
Nearest Centroid (NC)	TF-IDF	80%	*χ*^2^	*l*1	-	76.4%	2.9%
SGD	TF	55%	F	-	1/0.05	63.6%	1.0%
M-Naive Bayes (MNB)	TF-IDF	80%	F	-	20/80	64.1%	6.5%

T: Transformation; P: Percentile; FS: Feature Selection; N: Normalization; CW: Class_Weight / Prior Probability; SD: Standard Deviation

The objective of our study is to increase sensitivity in the search for infections, given that the manual procedure adopted by the surveillance team yields high precision and low sensitivity. The high sensitivity (and consequent low precision) searched in this study are depicted in the curves in Figs 4 and 7. Sensitivity was also prioritized here due to the fact that the gold-standard used in the analysis was based on sampling and selecting patients with higher potential risk; therefore, patients with lower risk that presented infection were less likely to have been investigated and accounted for in the gold-standard. Using text mining and machine learning to direct a more effective sampling by the surveillance team may lead to more infected patients being detected, yielding more reliable infection indicators and improving the gold-standard for future studies. Using the best algorithms for predicting and detecting SSIs may reduce the number of cases to be monitored in the post-operation period by more than 50% with less than 5% false negatives.

For predicting and detecting SSIs, the parameter CW (Class_Weight) was used to account for the unbalanced dataset, working similarly to a cost function with the objective of minimizing the bias between clean and infected classes. A CW = 1/0.01, for instance, implies in assigning a weight of 1 to a positive (infected) classification and 0.01 to a negative (clean) classification.

Oversampling and undersampling approaches were also tested to account for imbalance in the dataset (results are presented as S2 Supplement). We were unable to avoid model overfitting when using oversampling, regardless of optimizing hyperparameters. That did not occur when undersampling, however none of the tested algorithms was able to outperform results obtained through the Class_Weight method.

A reduction in the number of surgeries to be monitored in the post-operative period represents a gain in terms of cost reduction and personal involvement in SSI surveillance. To attain such benefits, we should look for a compromise between the reduction in the number of events to be surveilled and the number of false negatives (infected surgeries classified as clean). That is attained analyzing the ROC-AUC mean values of each classification method in predicting infections, and the confusion matrix associated with the best method.

The best ROC-AUC in Table 4 is given by the SGD method (mean = 79.70%), with confusion matrix displayed in Table 6. Adopting the SGD for prediction would lead in a reduction of 64.3% [= (*n* –TP–FP)/*n*] in the number of records to be analyzed by the ICIC, with (FP = ) 20 surgeries reported as clean but actually infected not surveilled (approximately 10% of all infected surgeries). On the other hand, if the objective was to reduce the incidence of false negative classifications, the best method would be Logistic Regression: with a reduction of 55.59% in the number of records to be analyzed by the ICIC, it would imply in only 4.26% false negatives. These numbers illustrate the potential of using TM and ML methods to rationalize SSI surveillance activities.

**Table 6:** SGD method for prediction–confusion matrix.

*n* = 15,479	Predicted clean	Predicted infected	
Actual clean	True negatives (TN): 9,930	False positives (FP): 5,352	15,291
Actual Infected	False negatives (FN): 20	True positives (TP): 168	188
	9,959	5,520	

## 4. Discussion

In this paper, we addressed patient safety surveillance through the use of text mining and machine learning methods using a database of surgical descriptions and post-operative follow-ups obtained from a high complexity University hospital. Our main goal was to establish the best TM and ML techniques for SSI prediction and detection using only textual data. For that, different methods of TM and ML were tested based on similar applications reported in the literature. Our results demonstrated that TM and ML are effective tools to support surveillance teams in the prediction and detection of SSIs, leading to improved patient care and safety.

Based on the TM and ML methods applied to our database of surgical descriptions it was possible to optimize surveillance efforts by reducing 55.59% of the volume of surgeries to be followed preventively, with only 4.26% of infections not detected using the Logistic Regression method. Using the SGD method, it was possible to reduce the volume of surgeries to be followed by 68.98%, although with a higher number of infections going undetected (10.64%). Independent of the ML method chosen, it is possible to optimize the time and resources invested in SSI surveillance, potentially increasing the number of SSIs that are currently undetected by the ICIC.

TM and ML methods rely on accurate classification of clean and infected surgeries by the surveillance team. Some factors may affect such classification, having a direct impact on the precision and sensitivity of TM methods. The identification of SSI requires interpretation of clinical and laboratory findings [52]. However, some surgical patients experience a short period (or no period) of hospitalization after surgery. The identification of patients developing infections after early discharge has been one of the challenges faced by infection surveillance methods [12].

The high unbalance in the dataset analyzed also imposes a challenge to the performance of TM and ML methods. The large number of negative SSI cases contributes to the increase in the false positive rate. We tried to compensate that using TM techniques combined with feature selection, TF-IDF transformation, and analysis of bigrams and trigrams. Another factor that contributes to increasing the number of false positives concerns the medical narrative described in the post-operative record, in which the patient is alerted to the risk of SSI. When constructing the bag of words, such alert may confuse classifiers since records of clean surgeries have terms that are usually related to the occurrence of infection. Alerting physicians about the impacts of misleading narratives on TM performance may help overcome the problem.

TM and ML methods have the potential to play an important role in adverse events’ surveillance, as pointed out in the literature [2], in the context of machine learning. Specifically regarding SSI, we demonstrated that TM and ML may be applied on reports created shortly after surgery to predict the occurrence of infections and on post-operative narrative records, to detect infections (and therefore develop preventive measures for future patients). The knowledge on SSI rates may be used as part of a feedback mechanism to decrease the future incidence of such infections [53], as well as in the training of ML algorithms in TM.

As future study we plan to explore the use of TM and ML to follow the post-operative records of specific groups of patients, selected by medical condition or age group, for example. We also view the use of additional information, such as examinations and prescriptions of medications available in the computerized system of the hospital, as potentially beneficial to improve the performance of TM and ML methods in the detection of SSIs. Finally, the literature dealing with the study of unbalanced datasets in text mining is constantly evolving. In our study, we followed the bootstrap strategy proposed by [54] to handle sample imbalance and tested binary classifiers suitable for unbalanced datasets; however, the study of alternative sample pre-treatment and classifiers is also a promising research direction.

## Supporting information

S1 Supplement Data and codes used in the analysis. (ZIP)

S2 Supplement Oversampling and undersampling approaches tested to account for imbalance in the dataset. (DOCX)
