# Supplementary material for: Predicting the occurrence of surgical site infections using text mining and machine learning
Source: PLoS One. 2019 Dec 13;14(12):e0226272. doi: 10.1371/journal.pone.0226272 (PMC6910696; doi:10.1371/journal.pone.0226272)
Supplement: S2 Supplement — (DOCX) [file pone.0226272.s002.docx]

# Supplemental material

In tables S1 to S3 we report results from balancing classes in the analysis. During the original analysis we tested oversampling and undersampling approaches. We could not avoid model overfitting when using an oversampling approach regardless of performing hyperparameters’ optimization. That did not occur when undersampling; however, no algorithm was able to outperform results obtained through the Class_Weight method. Tables S1 and S2 below give the undersampling and oversampling results in comparison with our adopted analytical strategy (Class_Weight); Table S3 gives the composition of the dataset used in each approach.

**Table S1**. Algorithms’ performances in predicting SSIs

|  | **Undersampling - ROC-AUC** | | **Class_Weight - ROC-AUC** | |
| --- | --- | --- | --- | --- |
|  |  |  |  |  |
| **Method** | **Mean** | **SD** | **Mean** | **SD** |
| Random Forest | 73.10% | 4.20% | 76.30% | 3.30% |
| Logistic Regression | 75.50% | 4.70% | 75.90% | 2.50% |
| LinearSVC | 72.90% | 5.60% | 79.00% | 4.70% |
| SVC | 71.50% | 5.90% | 75.30% | 4.60% |
| Nearest Centroid | 78.70% | 3.00% | 78.20% | 4.30% |
| SGD | 64.90% | 4.00% | **79.70%** | 3.30% |
| MultinomialNB | 77.60% | 3.00% | 75.00% | 4.60% |

**Table S2**. Algorithms’ performances in detecting SSIs

|  | **Undersampling - ROC-AUC** | | **Class_Weight - ROC-AUC** | |
| --- | --- | --- | --- | --- |
|  |  |  |  |  |
| **Method** | **Mean** | **SD** | **Mean** | **SD** |
| Random Forest | 73.70% | 5.10% | 76.10% | 3.40% |
| Logistic Regression | 69.10% | 2.40% | **80.60%** | 2.40% |
| LinearSVC | 73.80% | 5.70% | 78.10% | 2.50% |
| SVC | 79.40% | 4.80% | 61% | 6.30% |
| Nearest Centroid | 72.80% | 5.70% | 76.40% | 2.90% |
| SGD | 63.60% | 7.50% | 63.60% | 1% |
| MultinomialNB | 77.90% | 3.70% | 64.10% | 6.50% |

**Table S3**. SSI database analyzed with undersampling and oversampling approaches

| **Description** | **Prediction** | | | **Detection** | | |
| --- | --- | --- | --- | --- | --- | --- |
| **Undersampling** | **Infected surgeries** | **Clean surgeries** | **Total** | **Infected surgeries** | **Clean surgeries** | **Total** |
| Records used in the undersampled study | 188  (50%) | 188  (50%) | 376  (100%) | 202  (50%) | 202  (50%) | 404  (100%) |
| **Oversampling** | **Infected surgeries** | **Clean surgeries** | **Total** | **Infected surgeries** | **Clean surgeries** | **Total** |
| Records used in the oversampled study | 15,291  (50%) | 15,291  (50%) | 30,582  (100%) | 12,435  (50%) | 12,435  (50%) | 24,870  (100%) |
